# Validation of the Anhysteretic Magnetization Model for Soft Magnetic Materials with Perpendicular Anisotropy

**DOI:** 10.3390/ma7075109

**Published:** 2014-07-14

**Authors:** Roman Szewczyk

**Affiliations:** Institute of Metrology and Biomedical Engineering, Faculty of Mechatronics, Warsaw University of Technology, sw. A. Boboli 8, 02-525 Warsaw, Poland; E-Mail: szewczyk@mchtr.pw.edu.pl; Tel.: +48-22-234-8417; Fax: +48-22-849-0395

**Keywords:** magnetic anisotropy, magnetization processes, magnetic hysteresis

## Abstract

The paper presents results of validation of the anhysteretic magnetization model for a soft amorphous alloy with significant perpendicular anisotropy. The validation was carried out for the Jiles-Atherton model with Ramesh extension considering anisotropy. Due to the fact that it is difficult to measure anhysteretic magnetization directly, the soft magnetic core with negligible hysteresis was used. The results of validation indicate that the Jiles-Atherton model with Ramesh extension should be corrected to allow accurate modeling of the anhysteretic magnetization. The corrected model may be applied for modeling the cores of current transformers operating in a wide range of measured currents.

## 1. Introduction

Among commonly used models of magnetic hysteresis loop [1], Jiles-Atherton model is the most interesting one from the theoretical point of view. It is also one of the most useful models for technical applications [2], especially for development of PSpice models of inductive components [3]. However, the Jiles-Atherton model is based on the anhysteretic magnetization curve concept.

The anhysteretic magnetization curve can be determined experimentally, but this is very difficult from a technical point of view. In order to measure the anhysteretic magnetization for the given magnetizing field *H*, a sample of the soft magnetic material should be magnetized from the demagnetized state up to this field *H*, and then the local demagnetization by AC magnetic field biased by the field *H* should be carried out [4]. Such measurement is problematic due to the fact that the fluxmeter has to measure the flux density during demagnetization process. As a result of these difficulties, experimentally measured anhysteretic curve for anisotropic soft magnetic materials has not been presented in the literature yet.

This paper is filing this gap. Recently, the Magnetec Company introduced to the market a strongly anisotropic amorphous alloy with possibility of nanocrystallization, the NANOPERM LM (Fe_73.5_Cu_1_Nb_3_Si_15.5_B_7_) for current transformers (core M-391). This material exhibits very low coercive field. As a result, the hysteresis of this material can be neglected, and its measured magnetization loop reduces to the anhysteretic magnetization. On the basis of the conducted experiments, a previously presented model of anhysteretic magnetization can be validated.

## 2. Anisotropy of Ferromagnetic Materials

Due to the lack of crystalline structure, magnetocrystalline anisotropy energy is absent in amorphous alloy-based soft magnetic materials. Thus there are only three main sources of anisotropy in the amorphous magnetic materials [5]:

- shape of the magnetic sample (shape anisotropy),
- annealing in the magnetic field,
- mechanical stresses in the core.

In technical applications, the shapes and sizes of magnetic cores are standardized, so the shape-induced anisotropy energy can be neglected. 

After rapid quenching process, the anisotropy may be introduced to the sample during the thermal annealing of amorphous alloy in the presence of a magnetic field. It should be highlighted, that the easy axis of such anisotropy may be parallel or perpendicular to the direction of the ribbon, according to the direction of the magnetizing field applied during the annealing. On the other hand, the average anisotropy energy *K*_an_ induced during magnetic field annealing has to be determined experimentally due to its strong dependence on amorphous alloy composition [6]. 

Mechanical stress induced anisotropy energy density *K*_an_(σ) is given by the following equation [7]:

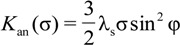
(1)
where λ*_s_* is the saturation magnetostriction and φ is the angle between the magnetizing field direction and the direction of the stress. However, one should note, that the saturation magnetostriction λ_s_ changes its value with applied mechanical stresses [8]. As a result, also in this case, the stress induced anisotropy energy density *K*_an_(σ) has to be determined experimentally for any given amorphous alloy composition.

## 3. Influence of Magnetic Anisotropy on Anhysteretic Magnetization in the Jiles-Atherton Model

Since its introduction in 1984, the Jiles-Atherton model [9] has been widely used for modeling the magnetic hysteresis loops of inductive components made of soft magnetic materials. The Jiles-Atherton model [9] is based on the idea of anhysteretic magnetization *M*_ah_. 

In the Jiles-Atherton model, the anhysteretic magnetization in the ferromagnetic materials is modeled similarly to the model of magnetization of paramagnetic materials [9,10]. In the case of paramagnetic materials, the value of magnetization *M*_para_ can be determined by considering the Boltzmann distribution of magnetic domain directions [11], given by following equation:

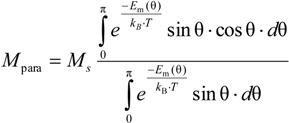
(2)
where *M*_s_ is the saturation magnetization of a paramagnetic material; θ is the angle between the atomic magnetic moment *m*_at_; and direction of the magnetizing field *H*; and *k_B_* is the Boltzmann constant. Energy of the magnetic moment *E*_m_(θ) is given as:

*E*_m_ (θ) = −μ_0_ · *m*_at_ · *H* · cosθ (3)

In the case of anhysteretic magnetization of isotropic ferromagnetic materials, the same Boltzmann distribution is used. In that case, the atomic magnetic moment *m*_at_ is substituted by the average magnetization of domain *m*_d_, given as [9]:

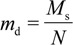
(4)
where *N* is the average domain density in the material. In the case of ferromagnetic materials, due to interdomain coupling described in the Jiles-Atherton model by the coefficient α, the effective magnetizing field *H*_e_ should be considered. This effective magnetic field is given by the equation [10]:

*H_e_* = *H* + *α · M* (5)

where α is interdomain coupling according to the Bloch model.

Considering Equations (3) and (4), the Boltzmann distribution based equation for anhysteretic magnetization (2) (and its antiderivative), leads to the Langevin equation for anhysteretic magnetization *M*_ah_iso_ of the ferromagnetic, isotropic materials [10]:

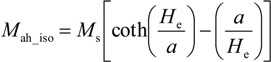
(6)
where *a* is given as:

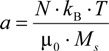
(7)


However, such simplified representation of the anhysteretic magnetization is valid only for isotropic materials. Due to the fact that most of recently developed materials are anisotropic, a special extension for model of anhysteretic magnetization was proposed [12]. This extension takes into account average anisotropy energy density *K*_an_ as well as direction of easy axis of magnetization. 

According to Ramesh *et al.* [13], for anisotropic materials, Equation (2) should be converted to anhysteretic magnetization in anisotropic magnetic materials *M*_ah_aniso_ [12,13]:

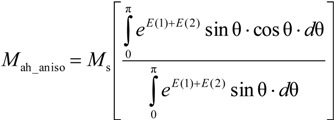
(8)
where

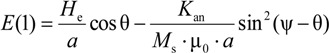
(9)

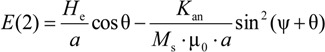
(10)


In such a case *K*_an_ is the average energy density connected with uniaxial anisotropy in a magnetic material, and ψ is the angle between direction of the magnetizing field and the easy axis of magnetization due to the anisotropy. It should be emphasized that functions in Equation (8) have no known antiderivatives. As a result, Equation (8) can be only solved using numerical integration.

On the other hand, this valuable concept of anhysteretic magnetization model for anisotropic materials has been not verified experimentally due to the lack of experimental results on measurements of the anhysteretic curves. Moreover, an editorial mistake was identified in Equation (8) presented by Ramesh *et al.* [13]. The presented form of Equation (8) is not consistent with the Langevin Equation (6) in the case of lack of average anisotropy energy (*K*_an_ = 0). Physical analysis of Equation (8) leads to the conclusion that its proper form is:

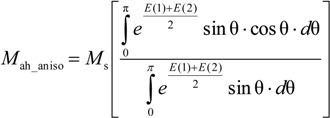
(11)


In such a case, consistence with the Langevin equation for *K*_an_ = 0 is achieved. 

Finally, the magnetic hysteresis loop is calculated from the following equation:

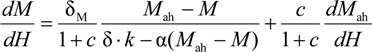
(12)
where the parameter *k* quantifies the average energy required to break pining site; and *c* describes reversibility of magnetization process. In this equation, parameter δ causes hysteretic magnetization and parameter δ_M_ guarantees that incremental susceptibility is always positive, which is physically justified [14].

**Figure 1:**
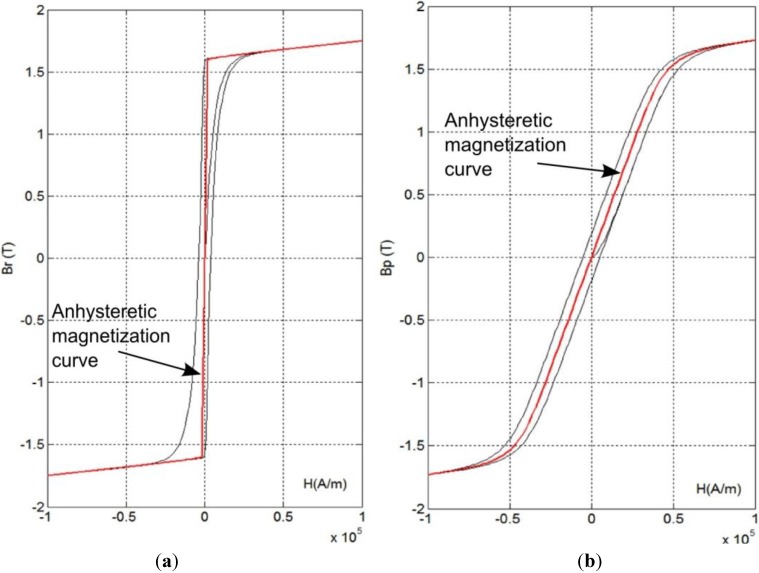
The anhysteretic magnetization curve and magnetic hysteresis loops of the anisotropic magnetic material with the Jiles-Atherton model’s parameters: *M*_s_ = 1.3 × 10^6^; *a* = 1000; α = 0.001; *k* = 5000; *c* = 0.1; *K*_an_ = 4 × 10^4^ for its magnetization (**a**) parallel to the easy axis (ψ = 0) (**b**) perpendicular to the easy axis (ψ = 90°).

Figure 1 presents the results of the anhysteretic magnetization curve modeling, as well as hysteresis loops for anisotropic material proposed by Ramesh *et al.* [13]. Modeling was carried out for easy axes of magnetization parallel (Figure 1a) and perpendicular (Figure 1b) to the magnetizing field direction. As it was expected, in both cases the anhysteretic magnetization curve is within the magnetic hysteresis loop of a material. However, a significant asymmetry of location of the anhysteretic magnetization within the hysteresis loop occurs for a material with easy axis parallel to the magnetizing field *H*.

## 4. Method of Measurements

The experiment was carried out using the M-391 core produced by the Magnetec GmbH (Langenselbold, Germany). The core had 30 mm outside diameter, 24.8 mm inside diameter and 6 mm of height. It was made of the NANOPERM LM (Fe_73.5_Cu_1_Nb_3_Si_15.5_B_7_) strongly anisotropic amorphous alloy with the possibility of nanocrystallization. The anisotropy was induced to the core during annealing under the influence of magnetic field.

**Figure 2:**
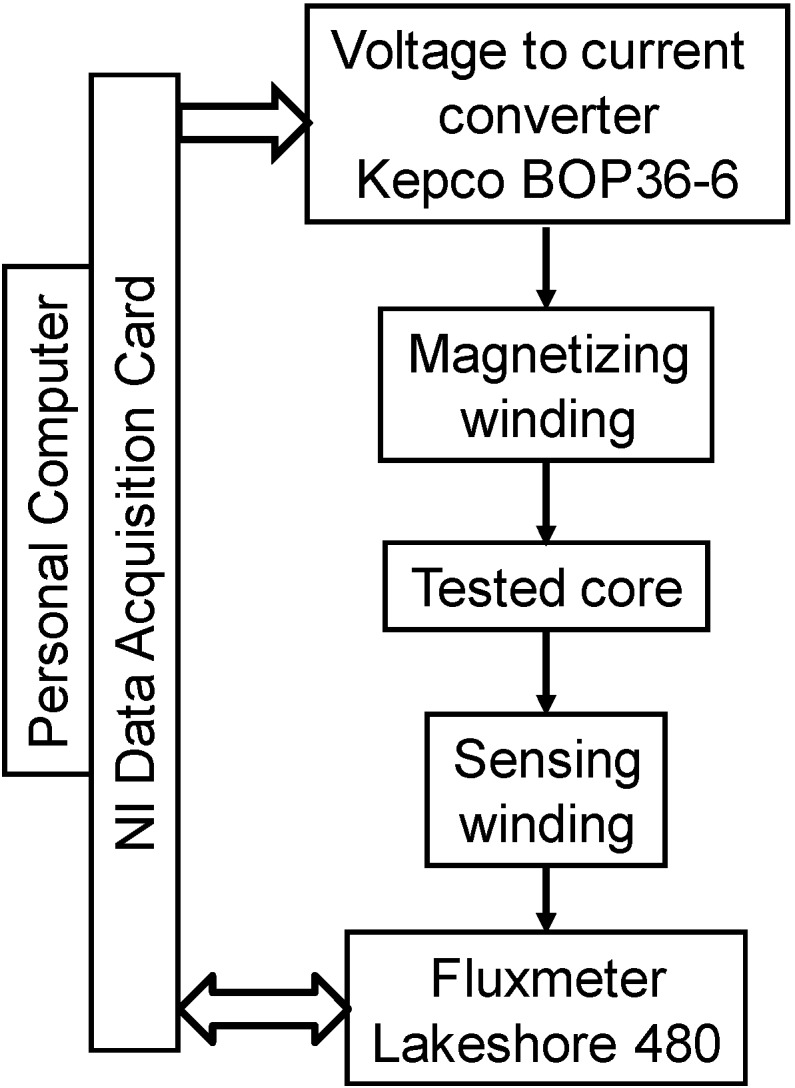
Block diagram of digitally controlled hysteresis graph.

Measurements of magnetic hysteresis loops were carried out with digitally controlled hysteresis graph presented in Figure 2. The magnetizing winding was connected to the output of the BOP36-6 high power voltage-current converter produced by Kepco Inc. (Flushing, NY, USA), whereas the sensing winding was connected to the input of the Model 480 fluxmeter produced by Lake Shore Cryotronics, Inc. (Westerville, OH, USA). The system was controlled by the personal computer equipped with data acquisition card produced by National Instruments (Austin, TX, USA). The measuring process was controlled by dedicated software developed in LabView environment.

## 5. Determination of the Jiles-Atherton Model Parameters

Parameters of the Jiles-Atherton model of the anhysteretic magnetization curve given by the Equation (11), were determined during the optimization process. The optimization was carried out from the point of view of minimization of the target function F given by the following equation:

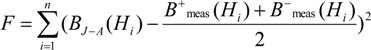
(13)
where *B_J-A_(H_i_)* were the results of the modeling of the anhysteretic curve at the point *H_i_*, whereas *B*^+^_meas_(*H_i_*) and *B*^−^_meas_(*H_i_*) are the results of measurements of the flux density *B* in the tested core respectively during the increase and decrease of magnetizing field. 

Determination of the model parameters by minimization of the target function *F* was performed with the use of simplex search method of Lagarias *et al.* [15]. Values of the determined parameters are presented in Table 1. 

**Table 1:** Jiles-Atherton model parameters for the anhysteretic curve of M-391 core.

Parameter	Quantity	Value
*a*	Parameter given by Equation (7)	2.066 A/m
α	Interdomain coupling	1.15 × 10^−12^
*K*_an_	Anisotropy energy density	417 J/m^3^
*M*_s_	Saturation magnetization	994,718 A/m

**Figure 3:**
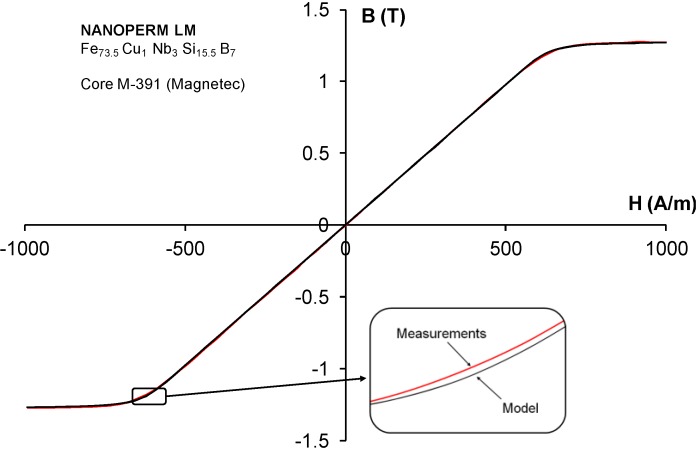
Results of measurements of *B*(*H*) dependency for the M-391 core with negligible hysteresis loop and modeled anhysteretic curve for this material.

Very good agreement between the results of anhysteretic magnetization of M-391 core modeling and the results of measurements of its *B*(*H*) dependency with negligible hysteresis loop are presented in Figure 3. It should be highlighted, that the anhysteretic magnetization covers *B*(*H*) dependency in the full range of magnetization *H*. Moreover, this very good agreement is confirmed by the value of *R^2^* determination coefficient which exceeds 0.99997. 

## 6. Discussion

In the case of materials with perpendicular anisotropy, value of average anisotropy density *K*_an_ can be estimated as the area of triangle between hysteresis loop for positive values of the magnetizing field H and y-axis [16]. As a result, the *K*_an_ value can be estimated from the following equation [17]:

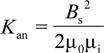
(14)
where μ_i_ is the relative initial permeability and *B*_s_ is the saturation flux density of a magnetic material. In the case of M-391 core, the initial permeability μ_i_ was 1555, whereas the saturation flux density *B*_s_ was 1.25 T. As a result, according to Equation (14), the average anisotropy energy density *K*_an_ may be estimated as 400 J/m^3^, which confirms results presented in the Table 1. Moreover, this good agreement between the two methods of average anisotropy energy density estimation confirms the correctness of Equation (11).

## 7. Conclusions

Due to the negligible hysteresis loop, NANOPERM LM (Fe_73.5_Cu_1_Nb_3_Si_15.5_B_7_-core M-391) strongly anisotropic amorphous alloy with the possibility of nanocrystallization creates new means of validation of the anhysteretic magnetization curve model. The conducted validation enabled correction of the anhysteretic magnetization curve in Jiles-Atherton model extended by Ramesh. After correction, a very good agreement between the results of anhysteretic magnetization of M-391 core modeling and results of its *B*(*H*) dependency measurements was observed. This very good agreement is confirmed by the value of *R*^2^ determination coefficient which exceeds 0.99997.
